# Identifying metabolic syndrome in migrant Asian Indian adults with anthropometric and visceral fat action points

**DOI:** 10.1186/s13098-022-00871-4

**Published:** 2022-07-15

**Authors:** John D. Sluyter, Lindsay D. Plank, Elaine C. Rush

**Affiliations:** 1 Section of Epidemiology and Biostatistics, Faculty of Medical and Health Sciences, University of Auckland, 28 Park Road, Auckland, 1023 New Zealand; 2 Department of Surgery, Faculty of Medical and Health Sciences, University of Auckland, Auckland, New Zealand; 3 School of Sport and Recreation, Faculty of Health and Environmental Sciences, Auckland University of Technology, Auckland, New Zealand

**Keywords:** Visceral fat, Diagnosis, Dual-energy X-ray absorptiometry, Metabolic syndrome, South Asian

## Abstract

**Background:**

Metabolic syndrome (MetS) is a clustering of metabolic risk factors, including large waist circumference (WC). Other anthropometric parameters and visceral fat mass (VFM) predicted from these may improve MetS detection. Our aim was to assess the ability of such parameters to predict this clustering in a cross-sectional, diagnostic study.

**Method:**

Participants were 82 males and 86 females, aged 20–74 years, of Asian Indian ethnicity. VFM was estimated by dual-energy X-ray absorptiometry (DXA) through identification of abdominal subcutaneous fat layer boundaries. Non-anthropometric metabolic risk factors (triglycerides, HDL cholesterol, blood pressure and glucose) were defined using MetS criteria. We estimated the ability of anthropometry and VFM to detect ≥ 2 of these factors by receiver operating characteristic (ROC) and precision-recall curves.

**Results:**

Two or more non-anthropometric metabolic risk factors were present in 45 (55%) males and 29 (34%) females. The area under the ROC curve (AUC) to predict ≥ 2 of these factors using WC was 0.67 (95% confidence interval: 0.55–0.79) in males and 0.65 (0.53–0.77) in females. Optimal WC cut-points were 92 cm for males (63% accuracy) and 79 cm for females (53% accuracy). VFM, DXA-measured sagittal diameter and suprailiac skinfold thickness yielded higher AUC point estimates (by up to 0.06), especially in females where these measures improved accuracy to 69%, 69% and 65%, respectively. Pairwise combinations that included WC further improved accuracy.

**Conclusion:**

Our findings indicate that cut-points for readily obtained measures other than WC, or in combination with WC, may provide improved detection of MetS risk factor clusters.

**Supplementary Information:**

The online version contains supplementary material available at 10.1186/s13098-022-00871-4.

## Introduction

Metabolic syndrome (MetS) prevalence has become an increasing problem among South Asian people [1, 2]. The definition of MetS is based on a cluster of metabolic risk factors: abdominal obesity, low HDL cholesterol, and raised glucose, triglycerides and blood pressure (BP). Criteria for identifying MetS in South Asian adults define abdominal obesity as a waist circumference (WC) of ≥ 90 cm in men and ≥ 80 cm in women [3].

A few studies have shown that WC can detect multiple metabolic risk factors relating to MetS in Asian Indians [4–7]. Most of these studies reported that this is also the case for waist:height ratio [5–8], waist:hip ratio [5–7] and BMI [5–8]. However, not all of these studies have specified appropriate cut-points for these anthropometric measures to identify these risk factor clusters, which limits their usefulness [6, 8]. These studies [4–8] did not compare the diagnostic performance of WC with that of visceral fat. Visceral fat may be more important than WC as it varies for a given WC and, compared to subcutaneous fat, is more closely related to metabolic risk factors [9–11]. However, quantification of visceral fat relies on measurement by instruments (such as CT or MRI) which are not accessible in many settings.

One approach is to use other measures of abdominal obesity, for example, sagittal diameter (of the abdomen) as multiple studies show that it is stronger correlate of visceral fat mass (VFM) and metabolic risk factors than WC and other anthropometric measures [12–15]. Another approach is to utilise VFM prediction equations based on accessible parameters as improvement in capturing VFM may translate into better detection of MetS clustering. A third approach is to combine these other parameters with WC when detecting clustering, which could increase confidence in diagnostic predictions [16]. However, to our knowledge, these approaches to detect MetS clusters have not been previously studied in Asian Indian people.

Given this knowledge gap, we investigated the ability of WC, other anthropometric parameters and VFM to identify clusters of MetS components in Asian Indian adults. We developed a prediction model for VFM based on its estimation from dual-energy x-ray absorptiometry (DXA) image analysis. We sought to provide appropriate cut-points for these metrics so that they may be used to define abdominal obesity for identifying MetS.

## Methods

### Participants

Participants were adults of Asian Indian ethnicity (self-identified) who resided in urban Auckland and were recruited by personal contact with community organisations. Exclusion criteria were: total knee or hip joint replacement, lifting weights more than once a week, pregnancy, major medical conditions (such as diabetes or cancer) and medication that could affect body composition (such as oral steroids). Participants were predominantly migrants (96% born overseas) who had lived in New Zealand for a median duration of 6 years (interquartile range: 4–13 years).

### Protocol

Participants arrived at the Body Composition Laboratory in the Department of Surgery, University of Auckland, after fasting overnight. Body weight and height were measured, a DXA scan was performed, BP (systolic (SBP) and diastolic (DBP)) was measured twice after lying for at least 15 min using a mercury sphygmomanometer and a stethoscope, and a venous blood sample was obtained.

### Measurements

Height (± 0.1 cm) and weight (± 0.1 kg) were measured with participants wearing light clothing or standard hospital gown and no shoes. An estimated clothing weight was subtracted. Using a non-stretch tape measure (Figure Finder Tape Measure, Novel Products Inc., Rockton, IL) with a device to ensure that constant tension was applied, WC (± 0.1 cm) was measured at the lateral mid-point between the lower rib and the iliac crest and hip circumference (HC; ± 0.1 cm) was measured at the maximum protusion of the gluteal muscles. Ratios based on these anthropometric measurements were calculated: waist:height ratio, conicity index {calculated as: ${\text{WC }}\left( {\text{m}} \right)/[0.109\sqrt {{\text{weight}}\;({\text{kg}})/{\text{height}}\;({\text{m}})} ]$}, BMI, waist:hip ratio and body adiposity index [calculated as: HC (cm)/height(m)^1.5^ − 18] [17, 18]. A portable, sliding-beam, abdominal caliper was used to measure anthropometric sagittal diameter (± 0.1 cm) at the largest supine anteroposterior diameter between the xyphoid process and the umbilicus at the end of normal expiration. For this measurement, data collection commenced in the middle phase of recruitment; data were thus available for a subset (n = 117) of participants only. Suprailiac skinfold thickness (± 0.1 mm) was measured with Harpenden calipers. Body composition (fat, fat-free soft tissue and bone mineral content) was measured by a pencil-beam DXA scanner (model DPX + with software version 3.6y, GE-Lunar, Madison, WI).

### DXA image analysis

To reduce inter-observer variability in data analysis, one person (J.D.S.) analysed all scans. An abdominal region of interest (ROI) box was formed from a horizontal line touching the iliac crest, a horizontal line at the junction of the T12 and L1 vertebrae, and lateral lines including the outline of the waist; these anatomical landmarks are similar to those used in DXA image analysis elsewhere [19]. In each box, fat mass (FM), fat-free soft tissue mass and bone mineral content derived from the DXA scan output were recorded.

To measure mass in a pixel box—a ROI box one pixel in height (0.96 cm; hereafter referred to in this paper as slice thickness) and width 0.48 cm—masses of larger boxes were subtracted (Additional file 1: Text S1). Although it was possible to use a slice thickness of 0.96 cm for image analysis, the advantage of being able to measure to this degree of thickness was offset by its lack of precision. That is, soft tissue mass was measured by DXA to the nearest gram, which was comparable to the total amount of mass (typically 3–6 g) in each pixel box. To overcome this problem, the slice thickness was increased to the height of the abdominal ROI box (bordered by the horizontal line touching the iliac crest and a horizontal line at the junction of the T12 and L1 vertebrae) so that rounding error was small (< 5%) compared to the mass analysed.

Along the ROI box (slice), strips (smaller boxes) of tissue 0.48 cm wide were analysed for mass and percent fat mass (%FM; calculated as 100 × FM/total mass). The borders of each box were adjusted to follow the contour of the abdomen [20]. Using x-coordinates of the boxes, the air:tissue boundary or outer boundary of the subcutaneous fat layer (SFL) on each side of the abdomen was identified as the junction between a box comprising no mass (air) and a neighbouring one consisting of mass (tissue). The inner boundary of the SFL was identified (by x-coordinates) as the point at which %FM difference between two adjacent boxes was greatest [21, 22]. Following this, a cross-section 1 cm thick was defined by averaging the x-coordinates of SFL boundaries and masses over the entire ROI box and dividing by 0.96 cm (pixel height). That is, analysis was performed in a cross-section that represents the “average” cross-section over the entire ROI box. This approach was chosen in order to provide a measure of VFM that is representative of total VFM and therefore total visceral adipose tissue (VAT) volume which is more strongly related to obesity-related metabolic risk factors than single-slice VAT area [23]. The quantity of FM in this cross-section is hereafter referred to as abdominal FM (AbFM).

The abdominal width or transverse external diameter (in cm) was calculated as the distance (± 0.48 cm) between the outer boundaries (left and right) within the cross-section. For each side, SFL width was calculated as the distance (± 0.48 cm) between the inner and outer SFL boundaries, and the average of the right and left sides was taken to obtain the subcutaneous fat width (in cm) [24]. Given that anthropometric sagittal diameter was not measured in all participants (30% missing), we quantified DXA-measured sagittal diameter, as described in Additional file 1: Text S2.

### Calculation of abdominal fat variables

VFM and subcutaneous FM (SFM) were quantified in a series of steps, as described in Additional file 1: Text S2. To normalise for height, AbFM, VFM and SFM were divided by the square of height, giving the ratios AbFM/height^2^, VFM/height^2^ and SFM/height^2^, respectively. Height^2^ has been shown to be an appropriate denominator for the adjustment of FM [25, 26]. To adjust for size, AbFM, VFM and SFM in the ROI box were divided by total mass in the same cross-section to give percent abdominal fat (%AbFM), percent visceral fat (%VFM) and percent subcutaneous fat (%SFM), respectively. We calculated visceral adipose tissue area (VAT_A_) and subcutaneous adipose tissue area (SAT_A_; Additional file 1: Text S2) and expressed the two as a ratio (VAT_A_/SAT_A_) [27].

### Blood biochemistry and metabolic syndrome

Blood serum and plasma samples were stored at – 85 °C and analysed in batches. Serum samples were assayed by Diagnostic Medlab Laboratories for triglycerides, high-density lipoprotein (HDL), low-density lipoprotein, total cholesterol and glucose. Lipids were measured by standard Roche-Hitachi methodology and HDL was by direct assay. Glucose was measured by the Roche Hitachi glucose oxidase method. All assays were within target limits specified by the RCPA Quality Assurance Program. Plasma samples were assayed by LabPlus of Auckland City Hospital for insulin levels using the Abbot Imx Insulin Assay (list No2A10, Abbot Laboratories, Japan). Insulin resistance (HOMA2-IR) and beta cell function (HOMA2-%B) were calculated using the homeostatic model assessment (HOMA) algorithm [28] derived from the fasting glucose and insulin levels (using a DOS algorithm supplied by Jonathan Levy, Oxford University).

MetS was identified using the International Diabetes Federation (IDF) criteria [3]. That is, MetS was defined as the presence of a large WC (≥ 90 cm in men and ≥ 80 cm in women) and at least two of the following: high triglycerides (≥ 1.69 mmol/L), low HDL (< 1.04 mmol/L in men and < 1.29 mmol/L in women), high BP (SBP ≥ 130 mmHg or DBP ≥ 85 mmHg or on BP medication) and high glucose (≥ 5.6 mmol/L) [3].

### Statistical analysis

Pairwise differences in continuous variables were assessed with the paired t-test. Group differences in participant characteristics were examined by the Student t-test or Mann–Whitney U test, as appropriate, for continuous variables and Fisher exact test for categorical variables. Multivariable linear regression was used to examine relationships between abdominal fat variables and metabolic risk factors, adjusted for age and sex. BMI, total body fat mass, smoking and alcohol were considered as additional covariates, but these did not significantly contribute to the models. Standardized regression coefficients were calculated to compare effect sizes of abdominal fat variables; these are unitless and thus allow the strength of associations with different variables to be directly compared [29]. The percent explained variance of each model was quantified from the multiple correlation coefficient (R^2^). Stepwise linear regression was used to develop prediction equations for VFM by sex. The potential predictors were WC, weight, height, waist:height ratio, conicity index, BMI, hip circumference, waist:hip ratio, body adiposity index and age [30, 31]. Each model was validated using leave-one-out cross-validation and the PRESS (predicted residual sum of squares) statistic was calculated to compare prediction accuracy between models. Linearity of associations and normality of the residuals of regression models were examined. Insulin, triglycerides, HOMA2-IR and HOMA2-%B were positively skewed and were logarithmically transformed. Variance inflation factors were examined to check for collinearity among independent variables in multivariable models.

Receiver operating characteristic (ROC) curves were constructed to determine the ability of VFM and WC to correctly classify individuals as having ≥ 2 non-adipose (non-WC) metabolic risk factors (IDF criteria for MetS). This MetS-based outcome excluded WC as it was used to develop cut-offs for abdominal obesity [5, 32]. We assessed discrimination performance with area under the ROC curve (AUC; with 95% confidence intervals) and diagnostic accuracy as the proportion of individuals that were correctly classified. For the ROC curves, optimal cut-points were based on maximum values of the Youden index (sensitivity + specificity—1). Cut-points based on maximising the product of sensitivity and specificity [33] were also provided. Further, we quantified sensitivity, specificity, positive predictive value (PPV), negative predictive value and F1 score. F1 score is the harmonic mean of sensitivity and PPV ranging from 0 to 100%, with a high score indicating low false positives and negatives [34]. We constructed precision-recall curves to show the association between PPV (precision) and sensitivity (recall) [34]. To summarize precision across sensitivities, we quantified area under these curves (range: 0–1), with higher area indicating better overall precision [34]. Contour plots of accuracy were constructed for pairwise combinations of thresholds for WC and non-WC parameters. For these, accuracy was assessed when both parameters exceeded their respective thresholds.

Data are presented as mean ± standard deviation (SD) unless stated otherwise. Statistical significance was set at P < 0.05 (2-sided). ROC and precision-recall curve analyses were performed using R version 3.6.3, and all other analyses were carried out using SAS version 9.4 (SAS Institute, Cary, NC).

## Results

Table 1 shows the characteristics of the participants (82 males and 86 females). Insulin variables and triglycerides were log-transformed for analysis as they were positively skewed. MetS prevalence was 40% in males and 29% in females. VAT_A_/SAT_A_ was substantially higher (P < 0.0001) in males (mean = 1.13 ± 0.45) than in females (mean = 0.61 ± 0.23). Within each sex, those with ≥ 2 metabolic risk factors (IDF MetS criteria) other than WC (45 males and 29 females) had more VFM, a larger WC, a higher BMI and a larger sagittal diameter (anthropometric and DXA-measured). Anthropometric and DXA-measured sagittal diameters were similar, with a pairwise (n = 117) mean difference (DXA-measured minus anthropometric) of –0.3 ± 2.0 cm (P = 0.09).

**Table 1:** Characteristics of participants^a^

Characteristic	Males (n = 82)		Females (n = 86)	
	Number of non-WC MetS risk factors^b^		Number of non-WC MetS risk factors^b^	
	< 2 (n = 37)	≥ 2 (n = 45)	< 2 (n = 57)	≥ 2 (n = 29)
Age (years)	44.1 ± 14.3	46.0 ± 11.8	42.4 ± 13.9	45.8 ± 11.6
Waist circumference (cm)	89.3 ± 7.7	95.6 ± 10.8*	84.0 ± 10.1	88.9 ± 9.1*
Large WC [n (%)]^c^	19 (51.4)	33 (73.3)*	38 (66.7)	25 (86.2)*
Waist:height ratio	0.53 ± 0.05	0.56 ± 0.07*	0.54 ± 0.07	0.57 ± 0.06*
Waist:hip ratio	0.93 ± 0.06	0.95 ± 0.06	0.83 ± 0.07	0.85 ± 0.08
Conicity index	1.28 ± 0.08	1.31 ± 0.07	1.23 ± 0.09	1.23 ± 0.09
Body mass index (kg/m^2^)	24.2 ± 2.9	26.3 ± 4.1*	25.3 ± 4.3	28.2 ± 4.8*
Body adiposity index (%)	25.8 ± 3.1	27.5 ± 4.7	33.4 ± 5.2	36.1 ± 6.2*
Suprailiac SFT (mm)	24.6 ± 11.6	31.8 ± 12.3*	28.6 ± 10.6	34.9 ± 10.2*
Thoracic height (cm)	21.6 ± 2.0	22.5 ± 2.8	20.8 ± 2.0	21.7 ± 1.7*
SD, anthropometric (cm)	21.6 ± 1.6	22.7 ± 2.5	20.6 ± 2.7	22.6 ± 2.8*
SD, DXA (cm)^d^	22.1 ± 2.3	24.0 ± 2.6	19.4 ± 2.5	21.3 ± 2.6*
Abdominal FM (g)	210.3 ± 74.7	280.9 ± 90.2*	241.2 ± 90.0	287.6 ± 87.6*
Visceral FM (g)	118.0 ± 47.3	156.7 ± 45.6*	98.5 ± 37.8	121.6 ± 36.0*
Subcutaneous FM (g)	92.3 ± 42.1	124.2 ± 55.1*	142.6 ± 61.9	166.0 ± 63.1
VAT_A_/SAT_A_ ratio	1.15 ± 0.53	1.11 ± 0.37	0.59 ± 0.23	0.64 ± 0.23
Fasting glucose (mmol/L)	5.1 ± 0.4	5.4 ± 0.5*	5.0 ± 0.4	5.4 ± 0.6*
Insulin (pmol/L)	70.3 (43.1–90.4)	78.9 (64.6–110.5)*	64.6 (47.4–86.1)	91.8 (71.8–128.4)*
Total cholesterol (mmol/L)	5.2 ± 0.9	5.6 ± 1.0	5.2 ± 1.0	5.5 ± 1.0*
LDL cholesterol (mmol/L)	3.4 ± 0.9	3.7 ± 1.1	3.2 ± 0.9	3.5 ± 0.9
HDL cholesterol (mmol/L)	1.3 ± 0.3	1.0 ± 0.2*	1.4 ± 0.3	1.1 ± 0.3*
Total: HDL cholesterol ratio	4.23 ± 0.91	5.96 ± 1.38*	3.72 ± 0.98	5.14 ± 1.17*
Triglycerides (mmol/L)	1.0 (0.9–1.3)	2.4 (1.8–2.9)*	1.0 (0.8–1.3)	1.9 (1.4–2.3)*
HOMA2-IR	1.32 (0.82–1.68)	1.53 (1.23–2.07)*	1.21 (0.89–1.52)	1.64 (1.38–2.35)*
HOMA2-%B	105.9 (84.3–130.6)	112.3 (89.5–138.9)	111.6 (80.8–130.5)	130.5 (85.3–166.0)
Systolic BP (mmHg)	114.7 ± 17.4	124.2 ± 18.3*	112.4 ± 20.3	117.6 ± 19.7
Diastolic BP (mmHg)	73.5 ± 8.8	77.9 ± 8.5*	71.1 ± 9.0	75.3 ± 8.7*
Antihypertensive drugs	0 (0)	2 (4.4)	0 (0)	0 (0)
Metabolic syndrome^e^	0 (0)	33 (73.3)*	0 (0)	25 (86.2)*

*BP* blood pressure; *DXA* dual-energy X-ray absorptiometry; *FM* fat mass; *HDL* high-density lipoprotein; *HOMA* homeostasis model assessment; *HOMA2-IR* insulin resistance; *HOMA2-%B* beta cell function; *LDL* low-density lipoprotein; *MetS* metabolic syndrome (IDF definition); *SAT*_*A*_ subcutaneous adipose tissue area (cm^2^); *SD* sagittal diameter; *SFT* skinfold thickness; *VAT*_*A*_ visceral adipose tissue area (cm^2^); *WC* waist circumference

^a^Values are mean ± standard deviation, median (interquartile range) or sample size (%)

^b^IDF criteria for MetS other than waist circumference

^c^IDF MetS criteria: ≥ 90 cm in males, ≥ 80 cm in females

^d^Data available for 57 males and 60 females

^e^IDF criteria (including waist circumference) for MetS

^*^P < 0.05 vs. < 2 non-WC MetS risk factors

### Associations between abdominal fat variables and metabolic risk factors

Table 2 shows multivariable-adjusted associations between abdominal fat variables and metabolic risk factors, with corresponding model R^2^ values listed in Additional file 1: Table S1. In separate models, VFM, SFM and WC were associated with all risk factors, except fasting glucose, total cholesterol and LDL cholesterol (Table 2). In most cases, associations were stronger for VFM than SFM and WC. When VFM and SFM were both in the model, they were independently associated with log_10_(insulin), log_10_(HOMA2-IR) and log_10_(HOMA-%B), with VFM having stronger relationships (maximum difference in standardized β: 0.129). In these models, VFM was negatively associated with HDL cholesterol (standardized β: − 0.312) and positively correlated with total/HDL cholesterol ratio and log_10_(triglycerides) (standardized β: 0.275 and 0.282, respectively), while no significant associations with SFM were seen. Similar patterns were observed when VFM and SFM were normalised for height (Additional file 1: Table S2). When VFM and SFM were corrected for total abdominal mass, similar patterns were observed for relationships with HDL cholesterol, total/HDL cholesterol and log_10_(triglycerides) (Additional file 1: Table S2).

**Table 2:** Standardized, adjusted^a^ regression coefficients for associations between abdominal fat variables and metabolic risk factors

Dependent variable	Number of abdominal fat variables in model				
	One^b^			Two^c^	
	VFM	SFM	Waist	VFM	SFM
Fasting glucose	0.138	0.154	0.102	0.059	0.121
log_10_(insulin)	**0.548**^‡^	**0.477**^‡^	**0.533**^‡^	**0.373**^‡^	**0.266**^†^
Total cholesterol	0.056	0.102	0.006	− 0.017	0.111
LDL cholesterol	0.118	0.107	0.048	0.076	0.065
HDL cholesterol	**− 0.313**^‡^	− 0.177*	**− 0.250**^†^	**− 0.312**^†^	− 0.001
Total/HDL cholesterol	**0.299**^‡^	0.192*	**0.208**^†^	**0.275**^†^	0.037
log_10_(triglycerides)	**0.306**^‡^	0.195*	**0.238**^†^	**0.282**^†^	0.036
log_10_(HOMA2-IR)	**0.550**^‡^	**0.480**^‡^	**0.532**^‡^	**0.372**^‡^	**0.270**^†^
log_10_(HOMA2-%B)	**0.451**^‡^	**0.376**^‡^	**0.460**^‡^	**0.323**^‡^	0.194*
Systolic blood pressure	0.155*	**0.194**^†^	**0.281**^‡^	0.042	0.171
Diastolic blood pressure	**0.344**^‡^	**0.369**^‡^	**0.412**^‡^	0.162	**0.278**^†^

*HDL* high-density lipoprotein; *HOMA* homeostasis model assessment; *HOMA2-IR* insulin resistance; *HOMA2-%B* beta cell function; *LDL* low-density lipoprotein; *SFM* subcutaneous fat mass; *VFM* visceral fat mass; *Waist* waist circumference

^a^Adjusted for age and sex

^b^VFM, SFM and waist circumference in separate models

^c^VFM and SFM in the same model

^*^P < 0.05, ^†^P < 0.01, ^‡^P < 0.001

### Prediction of metabolic risk factor groups

The ability of WC and other anthropometric variables to detect groups of ≥ 2 non-adipose metabolic risk factors for diagnosing MetS (IDF criteria) is summarized in Tables 3 (for males) and 4 (for females). This is shown also for VFM as measured by DXA and as predicted from WC, BMI and age in each sex. The regression coefficients, R^2^ and standard error of estimates of these prediction models are shown in Tables 3 and 4. In males (Table 3), the AUC point estimate was 0.67 for WC. In comparison, it was higher (although not significantly) for DXA-measured VFM (AUC = 0.73), DXA-measured sagittal diameter (AUC = 0.70) and suprailiac skinfold thickness (AUC = 0.68), while being similar for predicted VFM and BMI (both 0.67). In females (Table 4), the AUC point estimate was 0.65 for WC. In contrast, most other parameters yielded higher values: DXA-measured sagittal diameter (AUC = 0.70), suprailiac skinfold thickness (AUC = 0.69), predicted VFM (AUC = 0.69), BMI (AUC = 0.69), DXA-measured VFM (AUC = 0.68), anthropometric sagittal diameter (AUC = 0.68) and waist:height ratio (AUC = 0.68). Overall, most single risk factor groups (high triglycerides, low HDL and high glucose) contributed to the abovementioned improvements as the AUC for these was mostly higher for parameters other than WC (Additional file 1: Table S3). For example, the detection of high triglycerides in females improved when DXA-measured VFM was used to detect this (AUC = 0.70) instead of WC (AUC = 0.65).

**Table 3:** Discrimination performance of parameters to detect ≥ 2 metabolic risk factors^a^ excluding waist circumference in males

	AUC (95% CI)	P-value vs. waist	Threshold-specific metric					
			Cut-point^b^	Sensitivity (%)	Specificity (%)	PPV (%)	NPV (%)	Accuracy (%)
Waist	0.67 (0.55–0.79)	–	92 cm	60	68	69	58	63
Waist:height ratio	0.64 (0.52–0.76)	0.34	0.55	58	65	67	56	61
Waist:hip ratio	0.59 (0.46–0.72)	0.11	0.92	76	46	63	61	62
Conicity index	0.63 (0.51–0.76)	0.45	1.26	84	41	63	68	65
SD, DXA	0.70 (0.59–0.81)	0.40	23 cm	64	65	69	60	65
SD, anthropometric^c^	0.60 (0.45–0.75)	0.47	23.8 cm	32	93	82	59	63
Suprailiac SFT	0.68 (0.56–0.80)	0.86	30 mm	51	84	79	58	66
BMI	0.67 (0.55–0.78)	0.94	24 kg/m^2^	76	51	65	63	65
Body adiposity index	0.59 (0.47–0.72)	0.23	27.5%	44	78	71	54	60
VFM, DXA	0.73 (0.62–0.84)	0.25	125 g	76	76	69	67	68
VFM, predicted^d^	0.67 (0.55–0.79)	0.95	143 g	53	78	75	58	65

*CI* confidence interval; *DXA* dual-energy X-ray absorptiometry; *NPV* negative predictive value; *PPV* positive predictive value; *SD* sagittal diameter; *SFT* skinfold thickness; *VFM* visceral fat mass; *waist* waist circumference

^a^IDF criteria for metabolic syndrome other than waist circumference

^b^Based on maximum Youden index

^c^n = 57 (25 measurements missing)

^d^VFM (g) = 2.248 × WC (cm) + 4.441 × BMI (kg/m^2^) + 1.013 × Age (y) − 227.773 (R^2^ = 0.65, standard error of estimate = 30.1 g)

**Table 4:** Discrimination performance of parameters to detect ≥ 2 metabolic risk factors^a^ excluding waist circumference in females

	AUC (95% CI)	P-value vs. waist	Threshold-specific metric					
			Cut-point^b^	Sensitivity (%)	Specificity (%)	PPV (%)	NPV (%)	Accuracy (%)
Waist	0.65 (0.53–0.77)	–	79 cm	97	32	42	32	53
Waist:height ratio	0.66 (0.54–0.78)	0.59	0.50	93	33	42	90	53
Waist:hip ratio	0.56 (0.42–0.69)	0.11	0.90	24	88	50	69	66
Conicity index	0.53 (0.41–0.66)	0.02	1.22	55	56	39	71	56
SD, DXA	0.70 (0.59–0.82)	0.13	19 cm	62	72	53	79	69
SD, anthropometric^c^	0.68 (0.54–0.82)	0.44	21.8 cm	67	69	54	79	68
Suprailiac SFT	0.69 (0.58–0.81)	0.51	29 mm	69	63	49	80	65
BMI	0.69 (0.57–0.80)	0.40	24.5 kg/m^2^	83	51	46	85	62
Body adiposity index	0.62 (0.50–0.75)	0.66	32%	83	46	44	84	58
VFM, DXA	0.68 (0.57–0.80)	0.16	112 g	62	72	53	79	69
VFM, predicted^d^	0.69 (0.57–0.80)	0.48	95 g	86	49	46	88	62

*CI* confidence interval; *DXA* dual-energy X-ray absorptiometry; *NPV* negative predictive value; *PPV* positive predictive value; *SD* sagittal diameter; *SFT* skinfold thickness; *VFM* visceral fat mass; *waist* waist circumference

^a^IDF criteria for metabolic syndrome other than waist circumference

^b^Based on maximum Youden index

^c^n = 57 (25 measurements missing)

^d^VFM (g) = 1.415 × WC (cm) + 3.381 × BMI (kg/m^2^) + 0.599 × Age (y) − 129.803 (R^2^ = 0.68, standard error of estimate = 22.3 g)

**Fig. 1:**
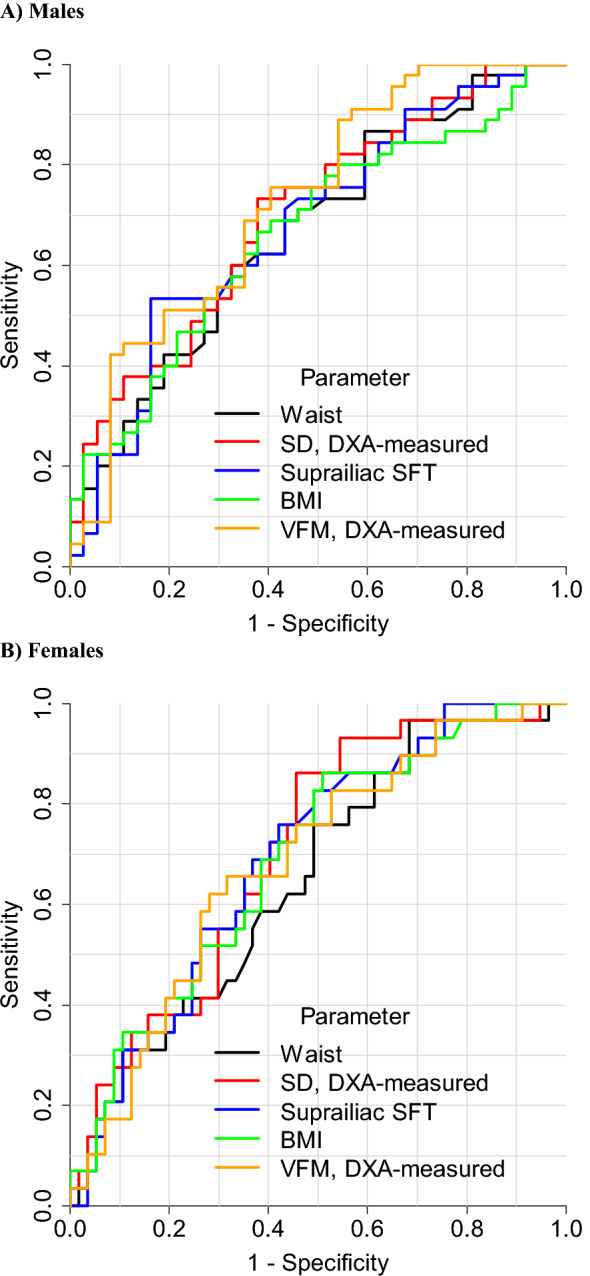
Receiver operating characteristic curves to detect ≥ 2 metabolic risk factors excluding waist circumference. *DXA* dual-energy X-ray absorptiometry; *SD* sagittal diameter; *SFT* skinfold thickness; *VFM* visceral fat mass; *Waist* waist circumference

**Fig. 2:**
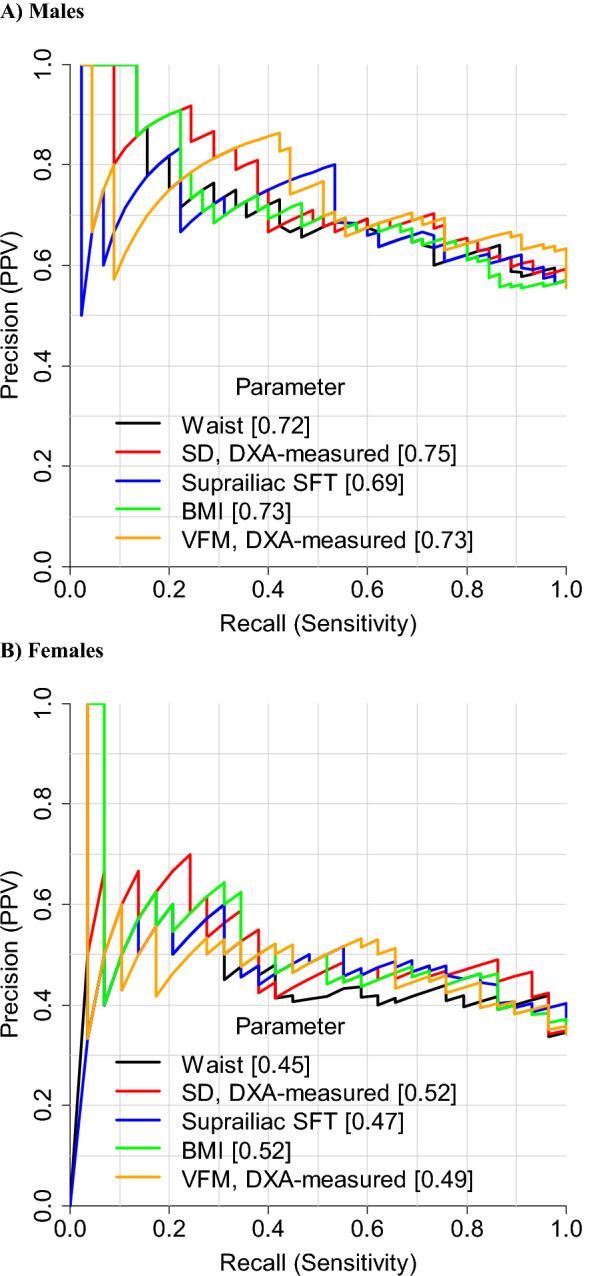
Precision-recall curves to detect ≥ 2 metabolic risk factors excluding waist circumference. *DXA* dual-energy X-ray absorptiometry; *SD* sagittal diameter; *SFT* skinfold thickness; *VFM* visceral fat mass; *Waist* waist circumference. Values in brackets give area under each curve

Tables 3 and 4 also show appropriate cut-points of parameters (based on maximum Youden index) to identify the presence of ≥ 2 non-adipose metabolic risk factors, along with diagnostic metrics. WC cut-points were 92 cm in males and 79 cm in females, with accuracy values of 63% and 53%, respectively. For males, accuracy was higher for most other parameters, especially DXA-estimated VFM, which had an accuracy of 68% at a cut-point of 125 g. This was true also among females, where accuracy was highest for DXA-measured VFM (69% at a cut-point of 112 g) and DXA-measured sagittal diameter (69% at a cut-point of 19 cm). ROC curves for parameters with higher AUC values compared to WC are shown in Fig. 1. PPV at a given sensitivity was higher overall for these parameters than for WC (Fig. 2). Cut-points for these parameters determined by maximising the product of sensitivity and specificity are provided in Additional file 1: Tables S4 and S5. These are very similar to those based on the Youden index except for WC in females which was 84 cm (rather than 79 cm) with higher accuracy (59 vs. 53%) and PPV (44 vs 42%).

**Fig. 3:**
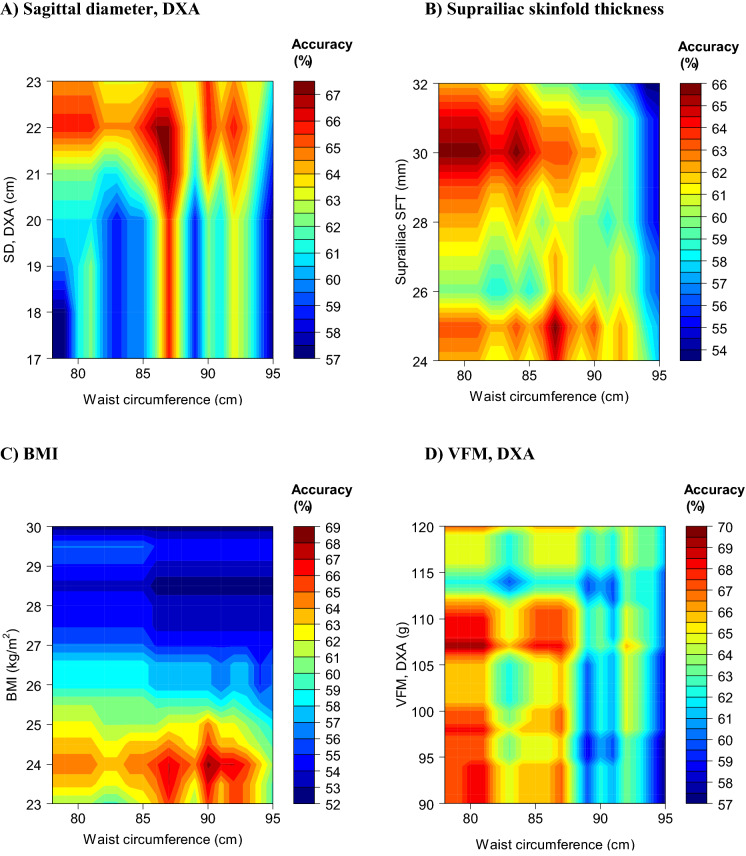
Accuracy to detect ≥ 2 metabolic risk factors excluding waist circumference with combinations of parameters: males. **A** Combination of waist circumference with DXA-measured sagittal diameter (SD). **B** Combination of waist circumference with suprailiac skinfold thickness (SFT). **C** Combination of waist circumference with body mass index (BMI). **D** Combination of waist circumference with visceral fat mass (VFM)

**Fig. 4:**
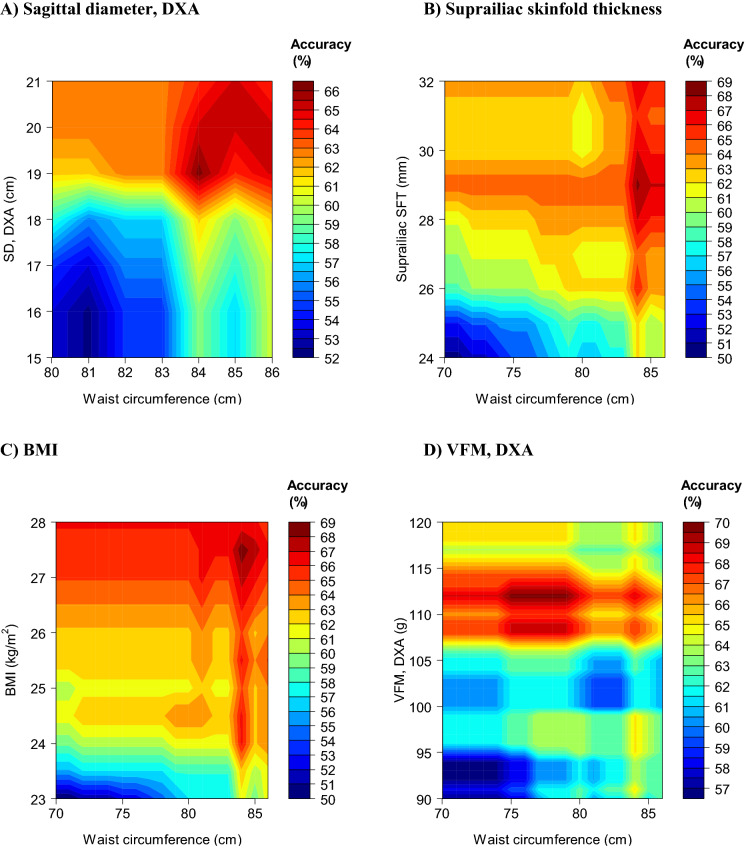
Accuracy to detect ≥ 2 metabolic risk factors excluding waist circumference with combinations of parameters: females. **A** Combination of waist circumference with DXA-measured sagittal diameter (SD). **B** Combination of waist circumference with suprailiac skinfold thickness (SFT). **C** Combination of waist circumference with body mass index (BMI). **D** Combination of waist circumference with visceral fat mass (VFM)

Next, for the same outcome, we examined whether combining WC with a non-WC parameter (when both had to exceed a certain threshold) improved diagnostic performance. We assessed performance for various combinations (pairs) of thresholds and determined which combination yielded the highest accuracy (Table 5, Figs. 3 and 4). Overall, using parameters in combination yielded higher accuracy and F1 score values (Table 5) than when these parameters were used separately (Additional file 1: Tables S4 and S5). For example, accuracy was highest (70%) when WC was combined with DXA-measured VFM. That is, when these 2 parameters had to exceed, in males, 78 cm and 107 g, respectively (F1 score = 77%) and, in females, 77 cm and 112 g, respectively (F1 score = 77%). In comparison, utilising these parameters separately achieved a lower maximum accuracy of 68% in males (F1 score = 75%) and 69% in females (F1 score = 57%).

**Table 5:** Optimal^a^ pairwise combinations of cut-points (including WC) to detect ≥ 2 metabolic risk factors^b^ excluding WC

Parameter	Cut-point		Threshold-specific metric					
	Parameter	WC (cm)	Sensitivity (%)	Specificity (%)	PPV (%)	NPV (%)	Accuracy (%)	F1 score (%)
Males								
SD, DXA	21 cm	87	84	46	66	71	67	74
Suprailiac SFT	25 mm	87	62	70	72	60	66	67
BMI	24 kg/m^2^	90	67	70	73	63	68	70
VFM, DXA	107 g	78	91	43	66	80	70	77
Females								
SD, DXA	19 cm	84	76	61	50	83	66	60
Suprailiac SFT	29 mm	84	57	74	52	77	68	55
BMI	27.5 kg/m^2^	84	45	81	54	74	69	49
VFM, DXA	112 g	77	62	74	55	79	70	58

*DXA* dual-energy X-ray absorptiometry; *NPV* negative predictive value; *PPV* positive predictive value; *SD* sagittal diameter; *SFT* skinfold thickness; *VFM* visceral fat mass; *WC* waist circumference

^a^Yielding the highest accuracy as determined from the analyses for Figs. 3 and 4

^b^IDF criteria for metabolic syndrome other than waist circumference

## Discussion

This study showed that, in Asian Indian adults, a WC of 92 cm for males and 79 cm for females were appropriate cut-points to identify the presence of ≥ 2 non-adipose metabolic risk factors associated with MetS. However, other parameters had a higher discrimination and precision performance, especially in females. These included VFM and sagittal diameter (both DXA-measured), suprailiac skinfold thickness and BMI. Combining these parameters with WC further improved diagnostic performance.

The discrimination performance of WC in detecting ≥ 2 non-adipose risk factors we observed for males (AUC = 0.67) is within the range of AUC values (0.627–0.94) for detecting MetS in Asian Indian males (urban) in previous studies [4–6]. Further, the WC threshold of 92 cm we deemed appropriate is similar to the 89 cm previously reported to identify clustering of MetS components in Asian Indian males [5] and the 90 cm to define abdominal obesity in MetS using modified NCEP-ATP III [35] and IDF criteria [36].

For females, in contrast, our AUC value of 0.65 for detecting ≥ 2 non-adipose metabolic risk factors with WC is lower than the 0.729–0.87 for urban females reported in previous Asian Indian studies [4–6]. As WC has differential associations with each MetS component (Additional file 1: Table S3), inter-study variations in the prevalence of these components in each MetS cluster may potentially contribute to this discrepancy. However, our suggested WC threshold for females of 79 cm is similar to the cut-points of 79–83 cm recommended in a previous study of Asian Indian adults [5] and 80 cm proposed by guidelines for the definition of MetS in this population [35, 36].

We extend the findings of these Asian Indian studies by comparing the discrimination performance of WC with that of VFM and other anthropometric indices not investigated in these studies (such as sagittal diameter, suprailiac skinfold thickness, conicity index and body adiposity index), providing cut-points for these, using precision-recall analyses (Fig. 2) and studying predominantly migrants who are exposed to nutrition-related acculturation effects (whereas prior studies comprised residents in India [4–6, 8]). Our finding that cut-points for sagittal diameter (DXA-measured and anthropometric) of ~ 23–24 cm in males and ~ 19–22 cm in females for detecting ≥ 2 non-adipose metabolic risk factors are close to the thresholds of ~ 24.6 cm (males) and ~ 22.5 cm (females) for this parameter (determined anthropometrically) for the detection of MetS in Mexican adults [37]. Consistent with this, prior adult studies of predominantly white populations reported appropriate thresholds of 22.2–26.9 cm (males) and 20.1–25.7 cm (females) for the detection of insulin resistance, dysglycemia, diabetes, elevated cardiovascular risk score and coronary heart disease [15, 38–41].

To our knowledge, this is the first study to examine the usefulness of VFM prediction equations based on anthropometry to detect MetS in South Asians. We found that the WC-based equations yielded an improvement in discrimination performance in females only, which may reflect that BMI is more indicative of body fat in females than in males [17]. Given that the predictors in these equations are simple to measure, our results support using these models for prediction of VFM and detection of MetS, although further confirmatory studies are required.

Another novel aspect of our study was the utility of combinations of two parameters that included WC (Table 5); illustrated using contour plots (Figs. 3 and 4). That we observed improvements in accuracy compared to using these parameters separately indicated that they provided non-redundant information about MetS clustering. In Chinese adults, combining BMI with WC was associated with a greater odds of cardiovascular risk factors than either alone [16]. This supports our findings, although we add to it by examining optimal combinations of cut-points, examining different diagnostic metrics and studying a different population.

Our findings can be used to screen for MetS more accurately. In practice, screening for MetS could start with WC as the first step since this is easy to measure. For this, the cut-points from Tables 3 and 4 may be used. Alternatively, to tailor to clinical preferences for the relative importance of false-positives and false-negatives, other cut-points are available for selection from Additional file 1: Tables S4 and S5. For example, for females, a WC cut-point of > 79 cm (Table 4) or, if there is preference for fewer false-positives, a higher threshold of > 84 cm (Additional file 1: Table S5) may be used. If WC is indicative of high risk of MetS (for example: > 92 cm in males, > 79 cm in females; Tables 3 and 4), the diagnosis of MetS would proceed with a second step comprising measurements of the remaining components of the MetS (triglycerides, HDL cholesterol, blood pressure and glucose [3]). To reduce diagnostic errors (false-positives and false-negatives), the first step could alternatively incorporate BMI measurements as we found that a combination of WC and BMI (males: WC > 90 cm and BMI > 24; females: WC > 84 cm and BMI > 27.5) improved diagnostic accuracy (Table 5). Further, our findings could also be applied in the research setting to more accurately screen for MetS in datasets using the anthropometric and VFM parameters assessed in our study.

A limitation of our study was that we did not use a reference method (CT or MRI) to measure VFM. However, the validity of our VFM measures is supported by our finding that metabolic risk factors were more strongly associated with VFM than SFM. This concurs with findings of previous studies which show that metabolic risk factors are more closely related to VAT than SAT measured with CT or MRI [9–11]. Secondly, in the DXA image analysis, we were restricted in our ability to obtain more accurate estimates of visceral fat as: (1) we were not able to measure mass in ROI boxes < 0.48 cm wide, which limited our ability to detect the edges of the SFL, and, (2) the ROI box precision (1 g) was comparable to the total amount of mass in each pixel box (typically 3–6 g), making it inappropriate to establish visceral fat area along every cross-section of the abdomen. Thus, these sources of measurement error may have attenuated the associations between VFM and metabolic risk factors. Thirdly, our models had imperfect accuracy. This could be due, not only to measurement error (as noted above), but to factors other than visceral fat that could impact on MetS. One factor, for example, could be skeletal muscle mass as it is a target for insulin and a reduction in this is associated with decreased insulin sensitivity in Asian Indian people [42]. Finally, statistical power to detect AUC differences was limited and a larger sample size may have allowed us to observe significant differences.

In conclusion, in Asian Indian adults, a WC of 92 cm for males and of 79 cm for females identified metabolic risk factor clusters and are appropriate for detecting abdominal obesity associated with MetS in Asian Indian populations. However, using other parameters (with cut-points deemed suitable) in the diagnosis improved detection; particularly when used in combination with WC. Longitudinal studies would help determine the prognostic usefulness of incorporating our proposed cut-points in the definition of MetS.

## Supplementary Information

**Additional file 1: Text S1.** Measurement of mass in a single pixel. **Text S2.** Calculation of abdominal fat variables. **Table S1.** Percent explained variance (R^2^; %) for models of adjusted associations between abdominal fat variables and metabolic risk factors. **Table S2.** Standardized regression coefficients for associations between abdominal fat variables and cardiometabolic risk factors, adjusted for sex and age. **Table S3.** Discrimination performance of parameters to detect metabolic risk factors other than waist circumference. **Table S4.** Diagnostic and predictive values of parameters to detect ≥2 metabolic risk factors (IDF criteria for MetS) other than waist circumference in males. **Table S5.** Diagnostic and predictive values of parameters to detect ≥ 2 metabolic risk factors (IDF criteria for MetS) other than waist circumference in females.

## Data Availability

Requests for the dataset analyzed for the current study can be made by contacting the corresponding author.
