# Supplementary material for: Identifying metabolic syndrome in migrant Asian Indian adults with anthropometric and visceral fat action points
Source: Diabetol Metab Syndr. 2022 Jul 15;14:96. doi: 10.1186/s13098-022-00871-4 (PMC9284905; doi:10.1186/s13098-022-00871-4)
Supplement: Supplementary file 1 — Additional file 1: Text S1. Measurement of mass in a single pixel. Text S2. Calculation of abdominal fat variables. Table S1. Percent explained variance (R2; %) for models of adjusted associations between abdominal fat variables and metabolic risk factors. Table S2. Standardized regression coefficients for associations between abdominal fat variables and cardiometabolic risk factors, adjusted for sex and age. Table S3. Discrimination performance of parameters to detect metabolic risk factors other than waist circumference. Table S4. Diagnostic and predictive values of parameters to detect ≥2 metabolic risk factors (IDF criteria for MetS) other than waist circumference in males. Table S5. Diagnostic and predictive values of parameters to detect ≥ 2 metabolic risk factors (IDF criteria for MetS) other than waist circumference in females. [file 13098_2022_871_MOESM1_ESM.docx]

**Supplementary material**

**Identifying metabolic syndrome in migrant Asian Indian adults with anthropometric and visceral fat action points**

John D. Sluyter, Lindsay D. Plank, Elaine C. Rush

**Abbreviations used in the Online Supplement:**

AbFM = abdominal fat mass; AUC = area under curve; AUPRC = area under precision-recall curve; DXA = dual-energy X-ray absorptiometry; NPV = negative predictive value; PPV: positive predictive value; MetS = metabolic syndrome; ROI = region of interest; SD = sagittal diameter; SFT = skinfold thickness; VFM = visceral fat mass.

**Contents**

**Text S1.** Measurement of mass in a single pixel **3**

**Text S2.** Calculation of abdominal fat variables **4**

**Table S1.** Percent explained variance (R^2^; %) for models of adjusted associations between abdominal fat variables and metabolic risk factors **7**

**Table S2.** Standardized regression coefficients for associations between abdominal fat variables and cardiometabolic risk factors, adjusted for sex and age **8**

**Table S3.** Discrimination performance of parameters to detect metabolic risk factors other than waist circumference **9**

**Table S4.** Diagnostic and predictive values of parameters to detect ≥2 metabolic risk factors (IDF criteria for MetS) other than waist circumference in males **10**

**Table S5.** Diagnostic and predictive values of parameters to detect ≥2 metabolic risk factors (IDF criteria for MetS) other than waist circumference in females **11**

**References 12**

**TEXT S1: Measurement of mass in a single pixel**

To measure mass in a single pixel (*Box 1*, as shaded in the image below*)* from a dual-energy X-ray absorptiometry scan image,

|  |  |  |
| --- | --- | --- |
|  |  |  |
|  |  |  |

*Box 1*

mass in a larger box that encompassed *Box 1* was firstly measured (*Box 2*). The row that *Box 1* was on was removed from *Box 2*, giving a third box (*Box 3*). Next, the column that *Box 1* was on was removed from *Box 2*, giving a fourth box (*Box 4*). Following this, the row *Box 1* was on was removed from *Box 4*, giving a fifth box (*Box 5*). Finally, mass in Box 1 was calculated as:

Mass in *Box 1* = (mass in *Box 2 –* mass in *Box 3*) *–* (mass in *Box 4* *–* mass in *Box 5*)

|  |  |  |
| --- | --- | --- |
|  |  |  |
|  |  |  |

|  |  |  |
| --- | --- | --- |
|  |  |  |
|  |  |  |

|  |  |  |
| --- | --- | --- |
|  |  |  |
|  |  |  |

|  |  |  |
| --- | --- | --- |
|  |  |  |
|  |  |  |

*Box 2* *Box 3* *Box 4* *Box 5*

**TEXT S2: Calculation of abdominal fat variables**

VFM and SFM were calculated from a series of steps involving several equations (see schematic diagram and table on next page):

TCA = mass/(density 🞨 ROI box height) (equation 1)

SD = (4 🞨 TCA)/(TED 🞨 π) (equation 2)

TID = TED – (2 🞨 SFW) (equation 3)

SID = SD – SFW (equation 4)

ICA = π(TID 🞨 SID)/4 (equation 5)

SFL area = TCA – ICA (equation 6)

SFM = (%FM 🞨 SFL area)/100 (equation 7)

VFM = AbFM – SFM (equation 8)

Schematic diagram of the abdomen (cross-section)*

|  |  |  |  |
| --- | --- | --- | --- |

|  |  |  |
| --- | --- | --- |

* Meaning of lines and areas in table:

| **Abbreviation** | **Meaning** | **Units** | **Color code in schematic diagram** |
| --- | --- | --- | --- |
| TCA | Total cross-sectional area | cm^2^ | \|  \| + \|  \| (both) \| \| --- \| --- \| --- \| --- \| |
| SD | Sagittal diameter | cm | \|  \|  \|  \|  \| \| --- \| --- \| --- \| --- \|   Note: Each yellow line = half of SFW (colour-coded blue) |
| TED | Transverse external diameter | cm | \|  \|  \|  \|  \| \| --- \| --- \| --- \| --- \| |
| TID | Transverse internal diameter | cm |  |
| SFW | Subcutaneous fat width  (average of left and right sides) | cm |  |
| SID | Sagittal internal diameter | cm |  |
| ICA | Internal cross-sectional area of an ellipse bordered by the inner boundary of the SFL | cm^2^ |  |
| SFL area | Area in the subcutaneous fat layer occupied by fat | cm^2^ |  |
| %FM | Average percentage fat content in the SFL on each side (right and left) of the abdomen |  |  |
| SFM | Subcutaneous FM | g |  |
| VFM | Visceral FM | g |  |

Equation 1 was applied separately for FM, fat-free soft tissue mass and BMC – using densities of 900 kg/m^3^, 1066 kg/m^3^ and 3317 kg/m^3^, respectively ([1](#_ENREF_1)) – and the three resultant area values were then summed to give TCA. Equation 2 uses the formula for calculating the area of an ellipse based on the length of its short and long axes, with evidence of very strong predictive performance (R^2^=0.98) ([2](#_ENREF_2)). In support of equations 3 and 4, studies have shown that that the product of TID and SD stripped of the SFL correlates strongly with ICA measured by MRI ([2](#_ENREF_2)) and with visceral adipose tissue (VAT) area measured by CT ([3](#_ENREF_3)). As SFW, rather than two times SFW, is used to estimate the SFL width in the same plane as the SD, equation 4 assumes that SFL width is thinner at the anterior and posterior regions (schematic diagram above). This assumption is consistent with what is observed anatomically ([2](#_ENREF_2)). In further support of this, Bertin *et al* ([4](#_ENREF_4)) subtracted different multiples of SFW from SD to measure SID and found that the highest correlation with VAT area (measured by CT) was obtained when SFW was subtracted.

After the final step (equation 8), SFM and VFM were converted into subcutaneous adipose tissue area (SAT_A_, in cm^2^) and VAT area (VAT_A_, in cm^2^), respectively, by assuming that adipose tissue contains 80% fat and has a density of 0.9255 kg/L ([5-7](#_ENREF_5)). That is, SFM and VFM were each divided by the product of 0.9255, 0.8 and slice thickness (1 cm) to give SAT_A_ and VAT_A_, respectively.

**Table S1.** Percent explained variance (R^2^; %) for models of adjusted ^a^ associations between abdominal fat variables and metabolic risk factors

| Dependent variable |  | Number of abdominal fat variables in model | | | | |
| --- | --- | --- | --- | --- | --- | --- |
|  |  | One ^b^ | | |  | Two ^c^ |
|  |  | VFM | SFM | Waist |  | VFM and SFM |
| Fasting glucose |  | 6.4 | 7.0 | 5.8 |  | 7.2 |
| log_10_(insulin) |  | 24.2 | 21.5 | 24.5 |  | 28.1 |
| Total cholesterol |  | 8.2 | 8.9 | 8.0 |  | 8.9 |
| LDL cholesterol |  | 4.3 | 4.3 | 4.8 |  | 4.6 |
| HDL cholesterol |  | 23.0 | 18.3 | 20.7 |  | 23.0 |
| Total/HDL cholesterol |  | 18.7 | 15.2 | 15.5 |  | 18.8 |
| log_10_(triglycerides) |  | 19.0 | 15.3 | 16.5 |  | 19.1 |
| log_10_(HOMA2-IR) |  | 24.2 | 21.6 | 24.3 |  | 28.3 |
| log_10_(HOMA2-%B) |  | 18.5 | 15.6 | 20.3 |  | 20.6 |
| Systolic blood pressure |  | 30.5 | 32.0 | 35.1 |  | 32.1 |
| Diastolic blood pressure |  | 20.7 | 23.7 | 25.6 |  | 25.0 |

HDL=high-density lipoprotein; HOMA=homeostasis model assessment; HOMA2-IR=insulin resistance;

HOMA2-%B=beta cell function; LDL=low-density lipoprotein; SFM=subcutaneous fat mass;

VFM=visceral fat mass; Waist=waist circumference.

^a^ Adjusted for age and sex. ^b^ VFM, SFM and waist circumference in separate models.

^c^ VFM and SFM in the same model.

**Table S2.** Standardized regression coefficients for associations between abdominal fat variables and cardiometabolic risk factors, adjusted for sex and age

| Dependent variable |  | Abdominal fat variable | | | | |
| --- | --- | --- | --- | --- | --- | --- |
|  |  | Type 1 models ^a^ | |  | Type 2 models ^a^ | |
|  |  | VFM/H^2^ | SFM/H^2^ |  | %VFM | %SFM |
| Fasting glucose |  | 0.050 | 0.121 |  | 0.018 | 0.108 |
| log_10_(insulin) |  | **0.395^‡^** | **0.210*** |  | **0.307^‡^** | **0.424^‡^** |
| Total cholesterol |  | -0.064 | 0.144 |  | 0.065 | 0.166 |
| LDL cholesterol |  | 0.033 | 0.084 |  | 0.129 | 0.155 |
| HDL cholesterol |  | **-0.278^†^** | 0.031 |  | **-0.261^†^** | -0.101 |
| Total/HDL cholesterol |  | 0.200 | 0.031 |  | **0.260^†^** | 0.162 |
| log_10_(triglycerides) |  | **0.286^†^** | 0.010 |  | **0.285^‡^** | 0.146 |
| log_10_(HOMA2-IR) |  | **0.393^‡^** | **0.214*** |  | **0.306^‡^** | **0.427^‡^** |
| log_10_(HOMA2-%B) |  | **0.348^‡^** | 0.140 |  | **0.277^†^** | **0.348^‡^** |
| Systolic blood pressure |  | 0.035 | 0.144 |  | 0.048 | **0.186*** |
| Diastolic blood pressure |  | 0.159 | **0.249*** |  | 0.126 | **0.311^‡^** |

%SFM=Percent subcutaneous fat; %VFM=Percent visceral fat; HOMA=Homeostasis model assessment; HOMA2-IR=Insulin resistance; HOMA2-%B=Beta cell function; LDL=Low-density lipoprotein; HDL=High-density lipoprotein; SFM=Subcutaneous fat mass (g); SFM/H^2^=Subcutaneous fat mass (g)/height (m)^2^; VFM=Visceral fat mass (g); VFM/H^2^=Visceral fat mass (g)/height (m)^2^.

^a^ Both abdominal fat mass variables are included in models.

*P<0.05, ^†^P<0.01, ^‡^P<0.001.

**Table S3.** Discrimination performance of parameters to detect metabolic risk factors ^a^ other than waist circumference

|  | | Males | |  | Females | |
| --- | --- | --- | --- | --- | --- | --- |
|  |  | AUC (95% CI) | P-value vs. Waist |  | AUC (95% CI) | P-value vs. Waist |
| High triglycerides (≥1.69 mmol/L) | n for high-risk group | n=40 |  |  | n=23 |  |
|  | Waist | 0.58 (0.46-0.71) | - |  | 0.65 (0.52-0.78) | - |
|  | SD, DXA-measured | 0.62 (0.50-0.74) | 0.32 |  | 0.67 (0.55-0.80) | 0.53 |
|  | Suprailiac SFT | 0.58 (0.46-0.71) | 0.98 |  | 0.66 (0.54-0.78) | 0.95 |
|  | BMI | 0.58 (0.46-0.71) | 0.99 |  | 0.69 (0.57-0.81) | 0.39 |
|  | VFM, measured | 0.63 (0.50-0.75) | 0.38 |  | 0.70 (0.58-0.82) | 0.32 |
| Low HDL cholesterol ^b^ | n for high-risk group | n=41 |  |  | n=41 |  |
|  | Waist | 0.60 (0.47-0.72) | - |  | 0.60 (0.48-0.72) | - |
|  | SD, DXA-measured | 0.66 (0.54-0.78) | 0.06 |  | 0.63 (0.51-0.75) | 0.56 |
|  | Suprailiac SFT | 0.63 (0.51-0.75) | 0.54 |  | 0.65 (0.53-0.76) | 0.51 |
|  | BMI | 0.63 (0.50-0.75) | 0.50 |  | 0.59 (0.46-0.71) | 0.70 |
|  | VFM, measured | 0.64 (0.52-0.76) | 0.32 |  | 0.59 (0.47-0.71) | 0.71 |
| High BP ^c^ | n for high-risk group | n=31 |  |  | n=22 |  |
|  | Waist | 0.63 (0.50-0.75) | - |  | 0.73 (0.61-0.84) | - |
|  | SD, DXA-measured | 0.58 (0.45-0.70) | 0.17 |  | 0.67 (0.54-0.81) | 0.24 |
|  | Suprailiac SFT | 0.58 (0.45-0.72) | 0.47 |  | 0.56 (0.43-0.69) | 0.02 |
|  | BMI | 0.57 (0.44-0.70) | 0.20 |  | 0.66 (0.53-0.79) | 0.22 |
|  | VFM, measured | 0.62 (0.49-0.74) | 0.82 |  | 0.73 (0.61-0.84) | 0.94 |
| High glucose (≥5.6 mmol/L) | n for high-risk group | n=24 |  |  | n=17 |  |
|  | Waist | 0.65 (0.50-0.79) | - |  | 0.64 (0.51-0.76) | - |
|  | SD, DXA-measured | 0.66 (0.52-0.80) | 0.79 |  | 0.71 (0.58-0.84) | 0.10 |
|  | Suprailiac SFT | 0.57 (0.42-0.73) | 0.21 |  | 0.63 (0.48-0.79) | 0.95 |
|  | BMI | 0.62 (0.47-0.76) | 0.42 |  | 0.71 (0.58-0.84) | 0.18 |
|  | VFM, measured | 0.60 (0.46-0.74) | 0.23 |  | 0.66 (0.53-0.79) | 0.74 |

BP = blood pressure; CI = confidence intervals; DBP = diastolic BP; SBP = systolic BP; SD = sagittal diameter; SFT = skinfold thickness; VFM = visceral fat mass; Waist = waist circumference. ^a^ IDF criteria for metabolic syndrome other than waist circumference. ^b^ <1.04 mmol/L in men, <1.29 mmol/L in women. ^c^ SBP ≥130 mmHg, DBP ≥85 mmHg or on BP medication.

**Table S4**. Diagnostic and predictive values of parameters to detect ≥2 metabolic risk factors (IDF criteria for MetS) other than waist circumference in males

| Parameter | Cut-point | Sensitivity  (%) | Specificity  (%) | PPV  (%) | NPV  (%) | Accuracy  (%) | F1 score  (%) | AUPRC |
| --- | --- | --- | --- | --- | --- | --- | --- | --- |
| Waist circumference (cm) | 86 | 87 | 32 | 61 | 67 | 62 | 72 | 0.72 |
|  | 87 | 87 | 41 | 64 | 71 | 66 | 74 |  |
|  | 88 | 80 | 41 | 62 | 63 | 62 | 70 |  |
|  | 89 | 73 | 41 | 60 | 56 | 59 | 66 |  |
|  | 90 | 71 | 51 | 64 | 59 | 62 | 67 |  |
|  | 91 | 62 | 59 | 65 | 56 | 61 | 64 |  |
|  | 92 | 60 | 68 | 69 | 58 | 63 | 64 |  |
|  | 93 | 58 | 68 | 68 | 57 | 62 | 63 |  |
|  | 94 | 51 | 70 | 68 | 54 | 60 | 58 |  |
| Sagittal diameter,  DXA (cm) | 19 | 100 | 11 | 58 | 100 | 60 | 73 | 0.75 |
|  | 20 | 98 | 16 | 59 | 86 | 61 | 73 |  |
|  | 21 | 87 | 32 | 61 | 67 | 62 | 72 |  |
|  | 22 | 82 | 46 | 65 | 68 | 66 | 73 |  |
|  | 23 | 64 | 65 | 69 | 60 | 65 | 67 |  |
|  | 24 | 40 | 78 | 69 | 52 | 57 | 51 |  |
|  | 25 | 33 | 89 | 79 | 52 | 59 | 47 |  |
|  | 26 | 20 | 97 | 90 | 50 | 55 | 33 |  |
|  | 27 | 13 | 97 | 86 | 48 | 51 | 23 |  |
| Suprailiac skinfold thickness (mm) | 24 | 73 | 49 | 63 | 60 | 62 | 68 | 0.69 |
|  | 25 | 69 | 57 | 66 | 60 | 63 | 67 |  |
|  | 26 | 62 | 57 | 64 | 55 | 60 | 63 |  |
|  | 27 | 58 | 65 | 67 | 56 | 61 | 62 |  |
|  | 28 | 53 | 73 | 71 | 56 | 62 | 61 |  |
|  | 29 | 53 | 76 | 73 | 57 | 63 | 62 |  |
|  | 30 | 51 | 84 | 79 | 58 | 66 | 62 |  |
|  | 31 | 49 | 84 | 79 | 57 | 65 | 60 |  |
|  | 32 | 44 | 84 | 16 | 55 | 62 | 56 |  |
| BMI (kg/m^2^) | 23 | 84 | 30 | 59 | 61 | 60 | 70 | 0.73 |
|  | 23.5 | 84 | 35 | 61 | 65 | 62 | 71 |  |
|  | 24 | 76 | 51 | 65 | 63 | 65 | 70 |  |
|  | 24.5 | 69 | 57 | 66 | 60 | 63 | 67 |  |
|  | 25 | 62 | 62 | 67 | 58 | 62 | 64 |  |
|  | 25.5 | 51 | 73 | 70 | 55 | 61 | 59 |  |
|  | 26 | 42 | 78 | 70 | 53 | 59 | 53 |  |
|  | 26.5 | 42 | 78 | 70 | 53 | 59 | 53 |  |
|  | 27 | 33 | 84 | 71 | 51 | 56 | 45 |  |
| VFM,  DXA (g) | 100 | 93 | 35 | 64 | 81 | 67 | 76 | 0.73 |
|  | 105 | 91 | 35 | 63 | 76 | 66 | 75 |  |
|  | 110 | 87 | 46 | 66 | 74 | 68 | 75 |  |
|  | 115 | 76 | 49 | 64 | 62 | 63 | 69 |  |
|  | 120 | 76 | 57 | 68 | 66 | 67 | 72 |  |
|  | 125 | 76 | 59 | 69 | 67 | 68 | 72 |  |
|  | 130 | 60 | 65 | 68 | 57 | 62 | 64 |  |
|  | 135 | 58 | 65 | 67 | 56 | 61 | 62 |  |
|  | 140 | 56 | 70 | 69 | 57 | 62 | 62 |  |
|  | 145 | 51 | 73 | 70 | 55 | 61 | 59 |  |

AUPRC=area under precision-recall curve; DXA=dual-energy X-ray absorptiometry; NPV=negative predictive value; PPV=positive predictive value; VFM=visceral fat mass.

Shaded cut-points are those that maximize the product of sensitivity and specificity ([8](#_ENREF_8)).

**Table S5**. Diagnostic and predictive values of parameters to detect ≥2 metabolic risk factors (IDF criteria for MetS) other than waist circumference in females

| Parameter | Cut-point | Sensitivity  (%) | Specificity  (%) | PPV  (%) | NPV  (%) | Accuracy  (%) | F1 score  (%) | AUPRC |
| --- | --- | --- | --- | --- | --- | --- | --- | --- |
| Waist circumference (cm) | 78 | 97 | 30 | 41 | 94 | 52 | 58 | 0.45 |
|  | 79 | 97 | 32 | 42 | 95 | 53 | 58 |  |
|  | 80 | 86 | 37 | 41 | 84 | 53 | 56 |  |
|  | 81 | 79 | 39 | 40 | 79 | 52 | 53 |  |
|  | 82 | 76 | 44 | 41 | 78 | 55 | 53 |  |
|  | 83 | 76 | 44 | 41 | 78 | 55 | 53 |  |
|  | 84 | 76 | 51 | 44 | 81 | 59 | 56 |  |
|  | 85 | 66 | 53 | 41 | 75 | 57 | 51 |  |
|  | 86 | 59 | 61 | 44 | 74 | 60 | 50 |  |
| Sagittal diameter,  DXA (cm) | 15 | 1 | 4 | 35 | 100 | 36 | 51 | 0.52 |
|  | 16 | 97 | 7 | 35 | 80 | 37 | 51 |  |
|  | 17 | 97 | 12 | 36 | 88 | 41 | 52 |  |
|  | 18 | 97 | 30 | 41 | 94 | 52 | 58 |  |
|  | 19 | 86 | 46 | 45 | 87 | 59 | 59 |  |
|  | 20 | 66 | 61 | 46 | 78 | 63 | 54 |  |
|  | 21 | 41 | 74 | 44 | 71 | 63 | 43 |  |
|  | 22 | 31 | 88 | 56 | 71 | 69 | 40 |  |
|  | 23 | 21 | 95 | 67 | 70 | 70 | 32 |  |
| Suprailiac skinfold thickness (mm) | 24 | 86 | 35 | 40 | 83 | 52 | 55 | 0.47 |
|  | 25 | 86 | 39 | 42 | 85 | 55 | 56 |  |
|  | 26 | 83 | 49 | 45 | 85 | 60 | 59 |  |
|  | 27 | 76 | 54 | 46 | 82 | 62 | 57 |  |
|  | 28 | 72 | 58 | 47 | 80 | 63 | 57 |  |
|  | 29 | 69 | 63 | 49 | 80 | 65 | 57 |  |
|  | 30 | 59 | 65 | 46 | 76 | 63 | 52 |  |
|  | 31 | 55 | 67 | 46 | 75 | 63 | 50 |  |
|  | 32 | 55 | 68 | 47 | 75 | 64 | 51 |  |
| BMI (kg/m^2^) | 23 | 90 | 30 | 39 | 85 | 50 | 55 | 0.52 |
|  | 23.5 | 86 | 40 | 42 | 85 | 56 | 57 |  |
|  | 24 | 86 | 46 | 45 | 87 | 59 | 59 |  |
|  | 24.5 | 83 | 51 | 46 | 85 | 62 | 59 |  |
|  | 25 | 72 | 54 | 45 | 79 | 60 | 55 |  |
|  | 25.5 | 69 | 60 | 47 | 79 | 63 | 56 |  |
|  | 26 | 59 | 65 | 46 | 76 | 63 | 52 |  |
|  | 26.5 | 52 | 70 | 47 | 74 | 64 | 49 |  |
|  | 27 | 48 | 74 | 48 | 74 | 65 | 48 |  |
| VFM,  DXA (g) | 88 | 83 | 40 | 41 | 82 | 55 | 55 | 0.49 |
|  | 92 | 76 | 47 | 42 | 79 | 57 | 54 |  |
|  | 96 | 76 | 54 | 46 | 82 | 62 | 57 |  |
|  | 100 | 66 | 58 | 44 | 77 | 60 | 53 |  |
|  | 104 | 66 | 60 | 45 | 77 | 62 | 54 |  |
|  | 108 | 66 | 68 | 51 | 80 | 67 | 58 |  |
|  | 112 | 62 | 72 | 53 | 79 | 69 | 57 |  |
|  | 116 | 48 | 74 | 48 | 74 | 65 | 48 |  |
|  | 120 | 45 | 75 | 48 | 73 | 65 | 46 |  |
|  | 124 | 45 | 79 | 52 | 24 | 67 | 48 |  |

AUPRC=area under precision-recall curve; DXA=dual-energy X-ray absorptiometry; NPV=negative predictive value; PPV=positive predictive value; VFM=visceral fat mass.

Shaded cut-points are those that maximize the product of sensitivity and specificity ([8](#_ENREF_8)).

**References**

1. Modlesky CM, Cureton KJ, Lewis RD, Prior BM, Sloniger MA, Rowe DA. Density of the fat-free mass and estimates of body composition in male weight trainers. J Appl Physiol. 1996;80:2085-96.

2. He Q, Engelson ES, Wang J, Kenya S, Ionescu G, Heymsfield SB, et al. Validation of an elliptical anthropometric model to estimate visceral compartment area. Obes Res. 2004;12:250-7.

3. Kvist H, Chowdhury B, Grangard U, Tylen U, Sjostrom L. Total and visceral adipose-tissue volumes derived from measurements with computed tomography in adult men and women: predictive equations. Am J Clin Nutr. 1988;48:1351-61.

4. Bertin E, Marcus C, Ruiz JC, Eschard JP, Leutenegger M. Measurement of visceral adipose tissue by DXA combined with anthropometry in obese humans. Int J Obes. 2000;24:263-70.

5. Kamel EG, McNeill G, Han TS, Smith FW, Avenell A, Davidson L, et al. Measurement of abdominal fat by magnetic resonance imaging, dual-energy X-ray absorptiometry and anthropometry in non-obese men and women. Int J Obes. 1999;23:686-92.

6. Kamel EG, McNeill G, Van Wijk MCW. Usefulness of anthropometry and DXA in predicting intra-abdominal fat in obese men and women. Obes Res. 2000;8:36-42.

7. Suliga E. Visceral adipose tissue in children and adolescents: a review. Nutr Res Rev. 2009;22:137-47.

8. Liu X. Classification accuracy and cut point selection. Stat Med. 2012;31:2676–86.
